# Different Responses of Ammonia-Oxidizing Archaea and Bacteria to Oxygen Depletion During Niche Separation in Water Column of Bohai Sea

**DOI:** 10.3390/biology15151280

**Published:** 2026-08-04

**Authors:** Ruotong Zhao, Yuqing Wang, Tianjiao Li, Ting Zhou, Xiaoxiao Guo, Gusheng Song, Liang Zhao, Jing Wang

**Affiliations:** 1 Tianjin Key Laboratory of Animal and Plant Resistance, Tianjin Key Laboratory of Conservation and Utilization of Animal Diversity, Tianjin Normal University, Tianjin 300387, China; 2 School of Marine Science and Technology, Tianjin University, Tianjin 300072, China; 3 College of Marine and Environmental Sciences, Tianjin University of Science and Technology, Tianjin 300457, China

**Keywords:** Bohai Sea, summer hypoxia, AOA, AOB, community structure

## Abstract

Summer hypoxia in the bottom water of the Bohai Sea is accompanied by marked changes in water-column structure and nutrient conditions, which may affect ammonia-oxidizing microorganisms and thus alter nitrification-related nitrogen cycling. To examine these responses, seawater samples were collected along an inshore–offshore transect from the coastal waters off Qinhuangdao City to the central Bohai Sea in June, July, and August 2018. Surface, middle, and bottom waters were sampled, and physicochemical variables, including temperature, salinity, dissolved oxygen (DO), and inorganic nitrogen concentrations, were measured. Ammonia-oxidizing archaea (AOA) and ammonia-oxidizing bacteria (AOB) were then analyzed using *amoA* amplicon sequencing to compare their diversity, community structure, genus-level composition, and dominant OTU patterns. From June to August, along with temperature increase, the water column showed stronger stratification and oxygen depletion in the bottom water, together with shifts in salinity and inorganic nitrogen distributions. AOA and AOB showed different temporal responses. AOA diversity surpassed that of AOB in July, but AOB became more diverse in August. Principal coordinates analysis showed clear separation of AOA communities between July and August, and sampling month had a significant effect on AOA community structure (PERMANOVA, *p* = 0.009). In contrast, AOB communities did not show significant month-related separation (*p* = 0.157). According to environmental fitting analysis, AOA community structure was predominantly associated with temperature and salinity, with only a marginal link to NH_4_^+^, while pH and salinity were the key factors shaping AOB communities. At the genus level, AOA was dominated by *Nitrosopumilus* and AOB was dominated by *Nitrosospira*. Overall, the results indicate that AOA showed stronger temporal variation than AOB during the development of summer hypoxia in seawater of Bohai Sea.

## Simple Summary

Seasonal oxygen depletion is becoming increasingly common in coastal seas and consequently alters the microorganisms that regulate nitrogen cycling. However, microorganisms performing the same ecological function may respond differently to these environmental changes. We investigated two major groups of ammonia-oxidizing microorganisms in the Bohai Sea during the development of oxygen depletion through summer. Seawater was collected from different locations and water depths in June, July, and August, and changes in environmental factors and microbial communities were examined. The two groups showed clearly contrasting patterns. Archaeal ammonia oxidizers underwent marked community changes between July and August, whereas bacterial ammonia oxidizers remained comparatively stable. Their community patterns were also associated with different environmental factors: the AOA was mainly related to temperature and salinity, while the AOB was more closely related to pH and salinity. These findings demonstrate that microorganisms responsible for the same step of nitrogen cycling do not necessarily respond in the same way to seasonal environmental change. Distinguishing these responses will improve our understanding of nitrogen cycling in oxygen-depleted coastal waters and help predict how ongoing eutrophication and ocean deoxygenation may affect marine ecosystem functioning.

## 1. Introduction

Coastal hypoxia is now recognized as a major environmental problem in marginal seas worldwide [1]. It is commonly driven by eutrophication, strengthened stratification, and enhanced oxygen consumption during organic matter decomposition. As oxygen declines, microbial nitrogen transformations can be strongly altered, with consequences for nutrient regeneration, nitrogen loss, and greenhouse-gas production [2]. Among these processes, nitrification is especially important because it links reduced and oxidized inorganic nitrogen pools and regulates substrate supply for denitrification and anaerobic ammonium oxidation [3,4]. The first step of nitrification, ammonia oxidation, is usually regarded as the rate-limiting step and is therefore critical for nitrogen cycling under oxygen-deficient conditions [5,6]. Recent work has further emphasized that microbial responses to deoxygenation are central to predicting changes in coastal nitrogen cycling [7].

Marine ammonia oxidation is mainly carried out by ammonia-oxidizing archaea (AOA) and ammonia-oxidizing bacteria (AOB), but these two groups do not respond to environmental change in the same way. In a wide range of marine habitats, AOA show greater abundance and wider distribution, whereas AOB generally inhabit narrower ecological niches [8,9]. Importantly, ammonia oxidation does not simply stop when oxygen becomes low. The existing literature has documented that marine AOA sustain activity during deoxygenation via specialized metabolic strategies for oxygen tolerance [10,11]. New evidence additionally reveals concurrent N_2_O production and consumption by marine AOA under oxygen depletion; meanwhile, differences in substrate utilization promote niche differentiation across dominant AOA lineages [12,13]. These observations reveal that AOA and AOB respond to different environmental constraints throughout hypoxia formation.

Unlike permanent open-ocean oxygen minimum zones, seasonal coastal hypoxia exhibits strong temporal variability, which is modulated by short-term changes, terrestrial nutrient inputs and intricate hydrodynamics [14]. These systems are therefore useful for examining how ammonia oxidizers respond to rapid environmental change. However, most prior studies on low-oxygen marine environments have focused on denitrification, anammox and total microbial communities, while the temporal turnover and environmental differentiation of planktonic AOA and AOB remain poorly understood [15,16]. Existing evidence from estuaries and coastal waters indicates that AOA are more sensitive to seasonal variations and environmental gradients than AOB. However, it remains unclear whether this pattern holds for semi-enclosed shallow seas with seasonal hypoxia [17].

The Bohai Sea in China is the only semi-enclosed shelf sea and has weak water exchange with the open ocean. In recent decades, recurrent summer hypoxia has been reported in the central Bohai Sea and has been linked to strong stratification, weak hydrodynamics, and enhanced oxygen consumption in bottom waters [18,19]. More recent studies indicate that stratification strength is a leading control on hypoxia formation in this region [20]. At the microbial level, hypoxia in the Bohai Sea has been shown to alter bacterial community composition, nitrogen-related pathways, and transcriptional activity. Metatranscriptomic evidence further suggests high transcriptional activity of Thaumarchaeota in deoxygenated seawater, including increased expression of genes related to ammonia oxidation, ammonia transport, and carbon fixation [21]. Despite these advances, direct evidence for how water-column AOA and AOB differ in composition, diversity, temporal turnover, and environmental responses during summer hypoxia development in the Bohai Sea still remains lacking [22,23].

In this study, we investigated AOA and AOB communities along an inshore–offshore transect from Qinhuangdao coastal waters to the central Bohai Sea during the progression of summer hypoxia. Based on *amoA* amplicon sequencing, we profiled the community composition and diversity of AOA and AOB among different stations, months and water layers, and subsequently investigated their associations with environmental variables. Specifically, we asked whether AOA and AOB showed different temporal and vertical distribution patterns during hypoxia formation, which environmental factor was most strongly associated with their community differentiation, and whether these differences indicated distinct ecological responses and niche partitioning under seasonal oxygen depletion. This study provides new evidence for understanding the microbial ecology of ammonia oxidation and its role in marine nitrogen cycling in seasonally hypoxic waters.

## 2. Materials and Methods

### 2.1. Study Area

Three cruises were conducted in the coastal waters off Qinhuangdao City and the central Bohai Sea on 16 June, 19 July, and 26 August 2018. During each cruise, five stations (H3–H7) were sampled along an inshore–offshore transect. The sampling stations were located at H3 (39.618° N, 119.840° E), H4 (39.478° N, 120.046° E), H5 (39.478° N, 120.252° E), H6 (39.408° N, 120.458° E), and H7 (39.377° N, 120.551° E). Stations H5–H7 were within the reported core hypoxic area from the northern Bohai Sea. At each station, seawater samples were collected from three depth layers, including surface, middle, and bottom waters (Table S1).

### 2.2. Seawater Sampling and Physicochemical Parameters Analysis

Sampling was carried out once in each month, yielding a total of 45 seawater samples. Seawater physicochemical parameters were measured in situ using a shipboard CTD (conductivity–temperature–depth) profiler (CTD; SBE 9plus, Sea-Bird Electronics, Bellevue, WA, USA). Temperature, salinity, dissolved oxygen (DO), and pH were recorded sequentially in the bottom, middle, and surface waters. Seawater samples were collected using 5 L Niskin bottles mounted on the CTD rosette, with 5 L of water collected from each layer. After retrieval, the samples were subsampled on deck using a sterilized bottle and kept on ice. After returning to shore, 1 L of seawater from each sample was filtered within up to 30 min onto a 0.22 μm polycarbonate membrane. The filtrations were applied in three replicates for each sample. In total, 3 L of seawater for each sample was collected on the membrane. The membranes were then folded, placed into sterile 2 mL cryovials, immediately preserved in liquid nitrogen, transported to the laboratory on dry ice, and stored at −80 °C until DNA extraction. The remaining 2 L of seawater was kept at 4 °C and transported to the lab on ice for nutrient analysis. Concentrations of ammonium, nitrate, and nitrite were determined using an AA3 HR Analyzer (SEAL Analytical, Mequon, WI, USA).

### 2.3. DNA Extraction and High-Throughput Sequencing

Prior to genomic DNA extraction via the commercial kit, three membranes from each sample were cut into fine fragments and mixed well. The FastDNA Spin Kit for Soil (MP Biomedicals, Irvine, CA, USA) was applied for DNA extraction according to the manufacturer’s instructions. Archaeal *amoA* genes were amplified with the primer set Arch-amoA-26F (5′-GACTACATMTTCTAYACWGAYTGGGC-3′) and Arch-amoA-417R (5′-GGKGTCATRTATGGWGGYAAYGTTGG-3′) [24], whereas bacterial *amoA* genes were amplified using amoA-1F (5′-GGGGTTTCTACTGGTGGT-3′)/amoA-2R (5′-CCCCTCKGSAAAGCCTTCTTC-3′) [25]. Each PCR was performed in duplicate in a final volume of 50 μL, containing 44 μL of 1.1 × T3 Super PCR Mix (TSINGKE, Beijing, China), 2 μL of each primer (10 μM), and 2 μL of template DNA [26]. The PCR program and reaction setup for each primer pair followed the corresponding published protocols [26].

Duplicate amplicons from the same sample were combined and subjected to paired-end sequencing on the Illumina MiSeq platform (Illumina, Inc., San Diego, CA, USA). Raw reads were first assigned to samples according to barcode and primer information, permitting no mismatches in barcodes and up to two mismatches in primer sequences. Quality control was carried out using fastp v0.20.0 [27]. Reads were trimmed with a 50 bp sliding window when the average base quality dropped below Q20, and reads shorter than 50 bp or containing ambiguous bases were discarded. Filtered paired-end reads were merged using FLASH v1.2.7 [28], requiring a minimum overlap of 10 bp and a maximum mismatch ratio of 0.2 within the overlap region. Reads that failed to satisfy these criteria were excluded from further analysis. Chimeric sequences were removed, and operational taxonomic units (OTUs) were clustered at 97% sequence similarity using UPARSE [29]. Representative OTU sequences were then taxonomically assigned against the NCBI non-redundant (NR) database using DIAMOND, followed by taxonomic annotation based on the lowest common ancestor (LCA) algorithm.

### 2.4. Data Analysis

Physicochemical variables were compared among sites and sampling times by a statistical significance test using one-way ANOVA in SPSS v17.0. The α-diversity calculations were based on evenly subsampled libraries. Shannon and Simpson indices were calculated to evaluate the diversity patterns of AOA and AOB communities in seawater samples.

To characterize community composition, OTU tables were transformed to relative abundance and summarized at the genus level. Genus-level community compositions of AOA and AOB were visualized as stacked bar plots. Differences in community structure among samples were assessed using Bray–Curtis dissimilarity matrices based on OTU relative abundance. Principal coordinates analysis (PCoA) was then performed to visualize the variation in AOA and AOB community structures among months and water layers. The significance of sampling month on community structure was tested by permutational multivariate analysis of variance (PERMANOVA).

Associations between community structure and environmental variables were further evaluated by environmental fitting analysis based on the PCoA ordination. The strength of association between each environmental variable and community structure was expressed as *R*^2^, and variables with *p* < 0.05 were considered significant, whereas 0.05 ≤ *p* < 0.10 was considered marginally significant. To further identify dominant taxa underlying temporal variation, the top 15 OTUs ranked by total abundance were selected separately for AOA and AOB. Heatmaps were generated based on row Z-scores of log-transformed relative abundance values to illustrate the standardized abundance patterns of dominant OTUs across samples. In these heatmaps, samples were ordered by month, station, and water layer to highlight month-dependent redistribution patterns of dominant OTUs.

## 3. Results

### 3.1. Physicochemical Properties of Bohai Seawater

**Figure 1:**
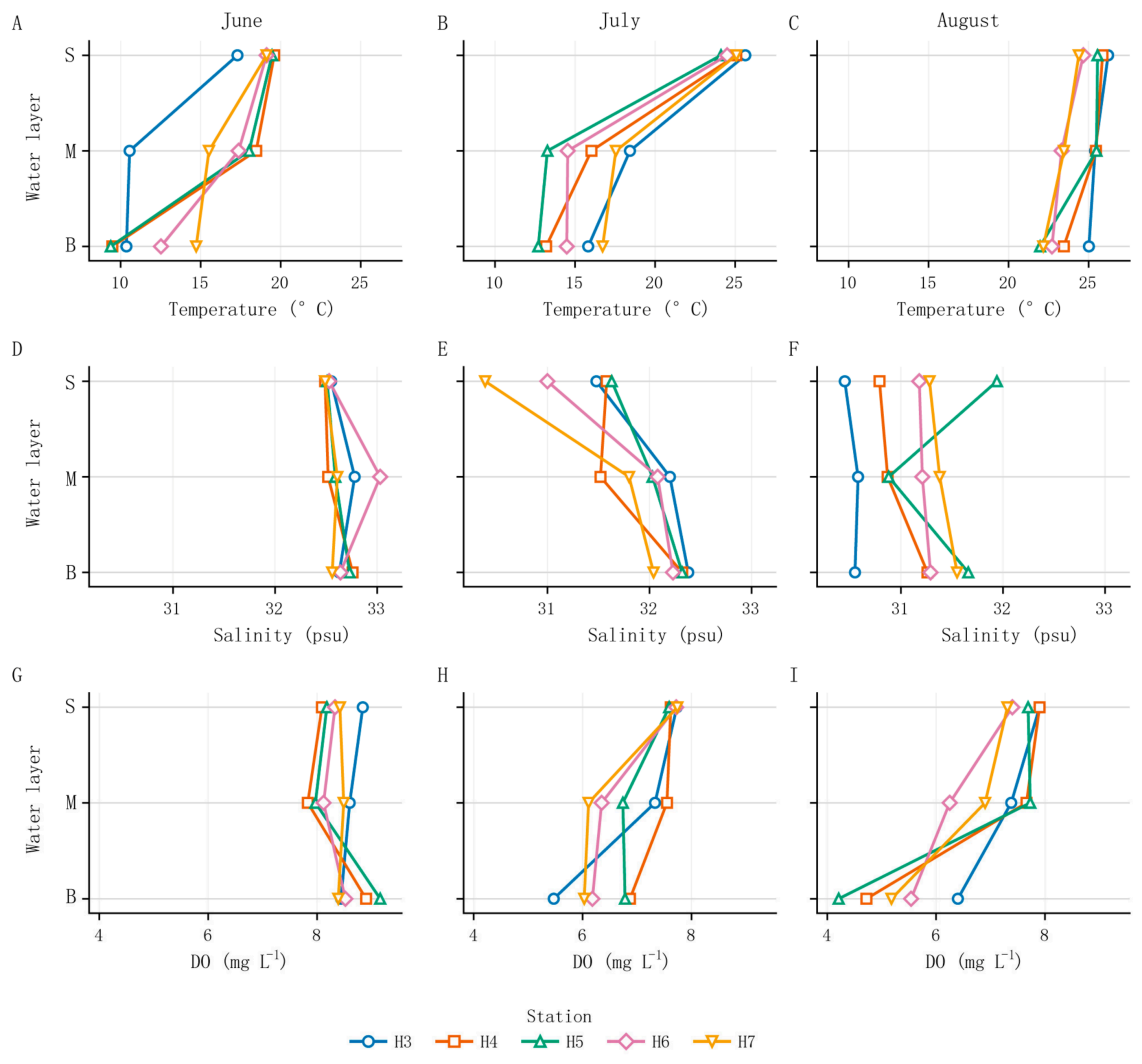
Vertical profiles of temperature, salinity, and dissolved oxygen (DO) in the water column across the Bohai Sea stations in June, July, and August. (**A**–**C**) show temperature, (**D**–**F**) show salinity, and (**G**–**I**) show DO. Within each panel, profiles are shown separately for stations H3–H7. S, M, and B indicate surface, middle, and bottom waters, respectively.

**Figure 2:**
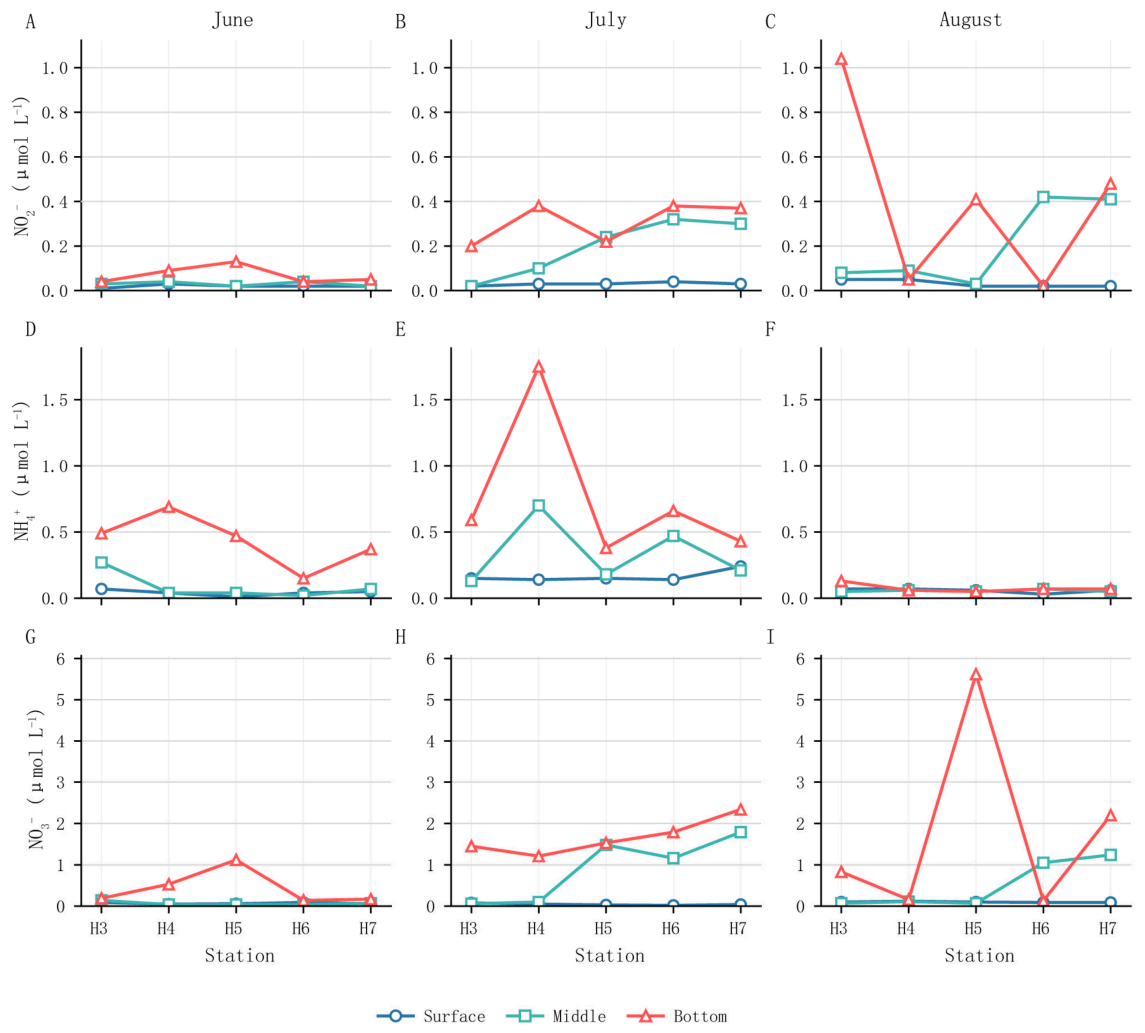
Layer-specific variations in inorganic nitrogen concentrations across the Bohai Sea stations in June, July, and August. (**A**–**C**) show NO_2_^−^ concentrations, (**D**–**F**) show NH_4_^+^ concentrations, and (**G**–**I**) show NO_3_^−^ concentrations. Concentrations are shown separately for surface (S), middle (M), and bottom (B) waters across stations H3–H7.

Overall, temperature, salinity, DO, and NH_4_^+^ concentrations were significantly different among months across the surface, middle, and bottom waters (*p* < 0.05). In bottom waters, temperature was significantly different between July and August (*p* < 0.01; Figure 1A–C). Significant differences in bottom-water salinity were mainly observed between August and June/July (*p* < 0.01; Figure 1D–F). Bottom-water DO concentrations were also significantly different between June and both July and August (*p* < 0.01; Figure 1G–I). In addition, bottom-water NH_4_^+^ concentrations were significantly different between July and August (*p* < 0.05; Figure 2D–F).

Along the vertical profile, the pattern of DO stratification varied among months. In June, no significant difference in DO concentration was found between the surface and bottom waters, whereas the middle and bottom waters were significantly different. In July, this pattern changed, with significant differences between the surface and middle waters and between the surface and bottom waters, while no significant difference was found between the middle and bottom waters. In August, the vertical pattern changed again: no significant difference was found between the surface and middle waters, whereas the bottom water was significantly different from both the surface and middle waters (Figure 1G–I). These results indicate that the vertical distribution of DO in the Bohai Sea changed markedly from June to August.

In addition, the three inorganic nitrogen species showed layer-specific variation among months and stations (Figure 2A–I). The patterns of NO_2_^−^, NH_4_^+^, and NO_3_^−^ were not consistent among the surface, middle, and bottom waters, indicating clear spatial heterogeneity. By comparison, temporal variation in NH_4_^+^ was more pronounced, particularly in bottom waters (Figure 2D–F).

### 3.2. Diversity of AOA and AOB in Bohai Seawater

High-throughput sequencing was applied to analyze AOA and AOB in seawater samples. Among the 45 samples, only 12 samples yielded valid sequence information for either one or both ammonia-oxidizing groups, including 10 AOA-positive samples and 9 AOB-positive samples, corresponding to 19 valid AOA/AOB sequencing datasets in total (Table S1). Successful sequencing was mainly obtained from samples collected in July and August, and most of these samples were from bottom waters. No PCR amplicons suitable for sequencing were obtained from the June samples, likely due to the low abundances of AOA and AOB. Detailed information on sequence numbers, normalized sequence numbers, OTUs, and observed species for each sample is provided in Table S1.

**Figure 3:**
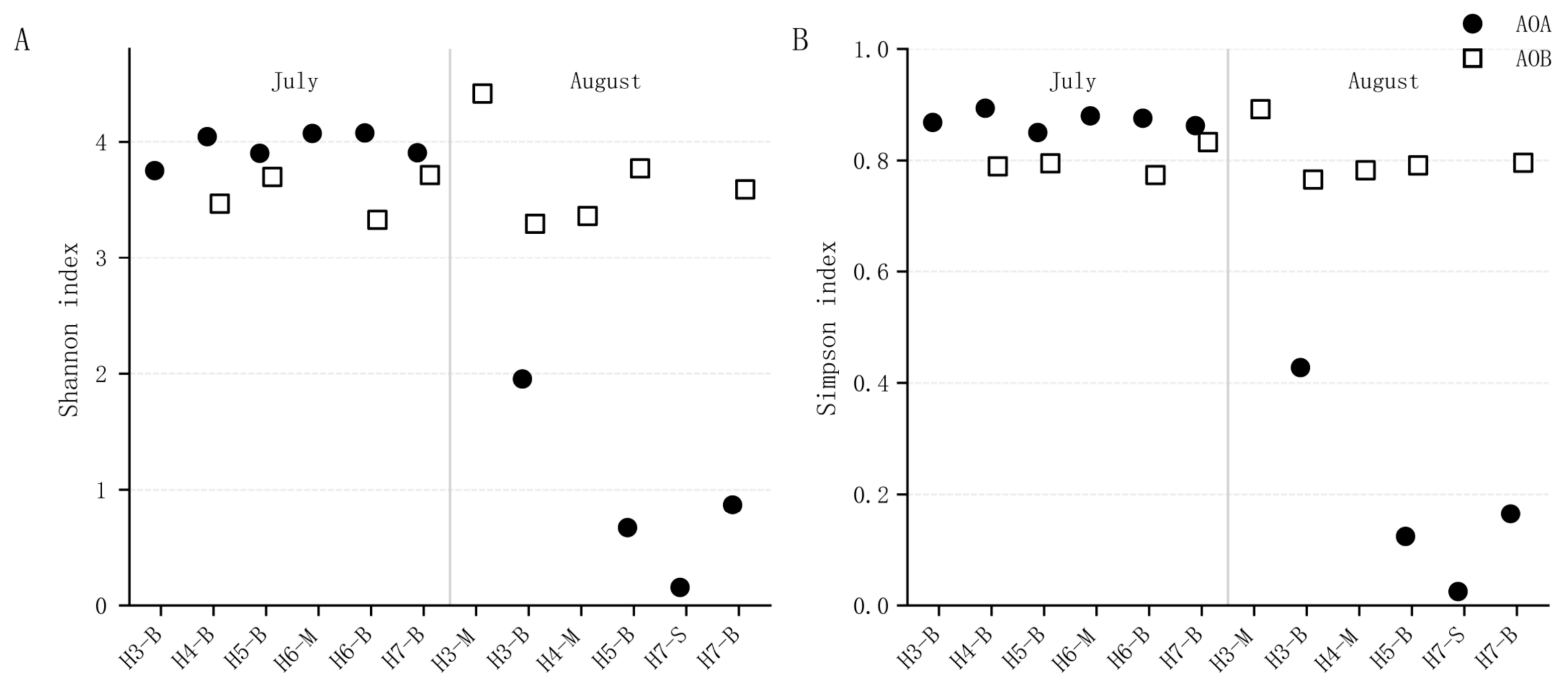
Alpha diversity of AOA and AOB in the Bohai Sea seawater samples. (**A**) shows the Shannon index and (**B**) shows the Simpson index. AOA and AOB are presented in the same coordinate system for direct comparison. Each point represents one sample, and overlapping points were slightly offset for clarity.

Shannon and Simpson indices showed similar patterns (Figure 3). In the July samples, AOA diversity was higher than AOB diversity, whereas in August, AOB diversity exceeded that of AOA. These results indicate that AOA diversity varied more markedly among months and was more strongly influenced by temporal variation. In contrast, AOB diversity changed less across months and appeared to be less sensitive to temporal variation. Therefore, AOB diversity was higher than AOA diversity in the August seawater samples.

### 3.3. Community Structure and Environmental Drivers of AOA and AOB in the Bohai Sea

**Figure 4:**
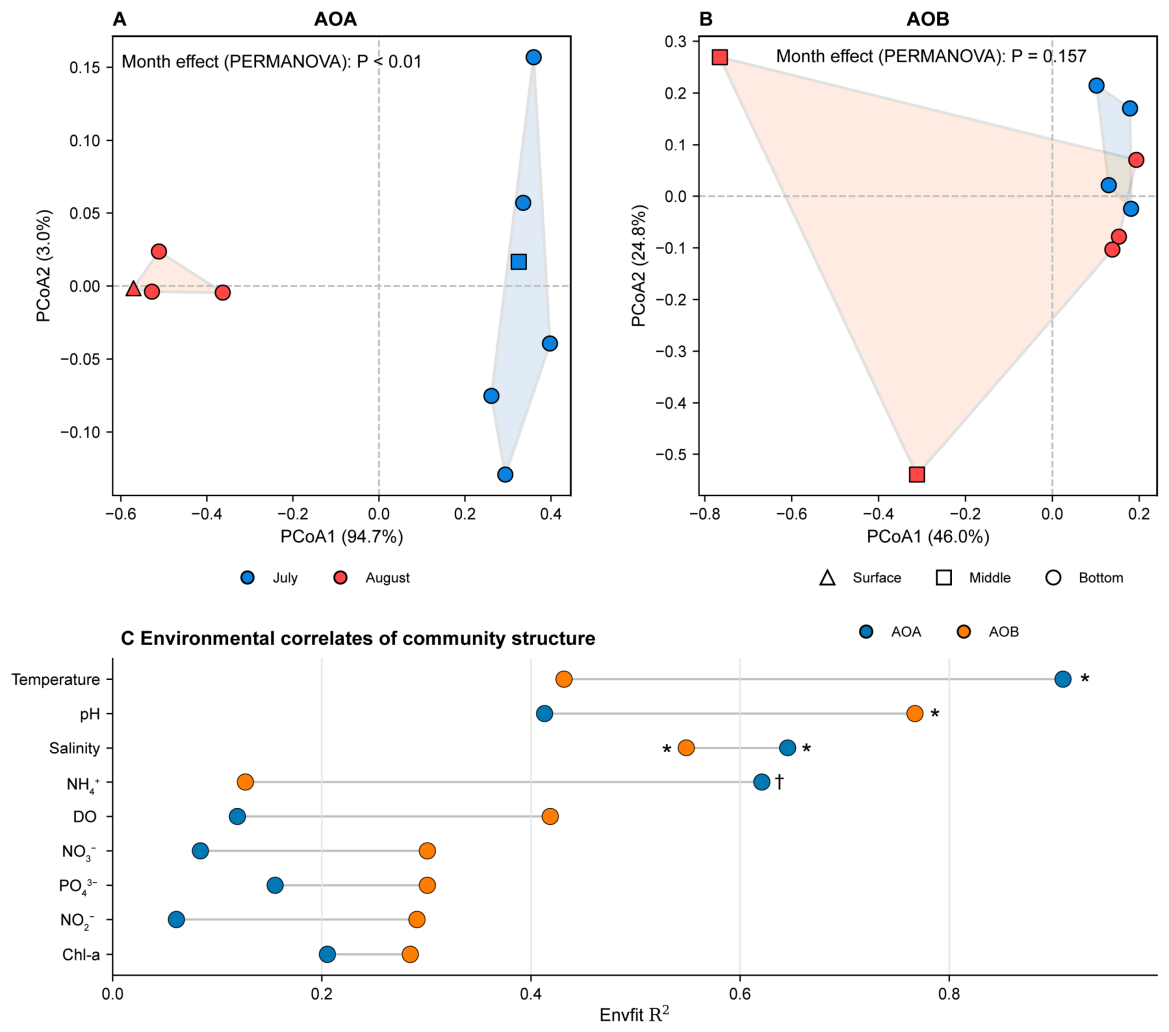
Principal coordinates analysis (PCoA) of AOA and AOB communities in Bohai Sea seawater samples. (**A**) shows the AOA community and (**B**) shows the AOB community. Colors indicate sampling month (July and August), and symbols indicate water layers (surface, middle, and bottom waters). The *p* values shown in each panel were obtained from PERMANOVA testing the effect of sampling month on community structure. (**C**) summarizes the relationships between environmental variables and AOA/AOB community structures. The positions of the points indicate the strength of association (Envfit *R*^2^), and asterisks and daggers indicate significant (*p* < 0.05) and marginally significant († 0.05 ≤ *p* < 0.10) associations, respectively.

PCoA analysis showed clear differences in community structure between AOA and AOB in response to sampling month. For AOA, samples collected in July and August were clearly separated along the first principal coordinate axis (Figure 4A), indicating a marked shift in community composition between the two months. Most July samples clustered on one side of the ordination space, whereas August samples clustered on the other side. PERMANOVA further confirmed that sampling month had a significant effect on AOA community structure (*p* = 0.009). The first two axes explained 94.7% and 3.0% of the total variation, respectively, suggesting that temporal variation was a major factor structuring the AOA community in seawater.

In contrast, the AOB samples did not show a clear separation between July and August in the PCoA ordination (Figure 4B). Samples from these two months were largely intermingled, and PERMANOVA showed that the effect of sampling time on AOB community structure was not significant (*p* = 0.157). The first two axes explained 46.0% and 24.8% of the total variation, respectively. Although a few August samples, particularly the middle-water samples from stations H3 and H4, were positioned away from the main cluster, the overall AOB community pattern did not show a consistent month-related separation.

Environmental fitting analysis showed that AOA and AOB community structures responded differently to environmental variables (Figure 4C). For AOA, temperature showed the strongest association with community structure and was significant, while salinity was also significantly associated with AOA community structure. In addition, NH_4_^+^ showed a marginal association. By contrast, pH, DO, NO_3_^−^, PO_4_^3−^, NO_2_^−^ and Chl-a were not significantly associated with AOA community structure. For AOB, pH and salinity were significantly associated with community structure, whereas temperature, NH_4_^+^, DO, NO_3_^−^, PO_4_^3−^, NO_2_^−^ and Chl-a showed no significant associations. Overall, AOA community structure was mainly associated with temperature and salinity, whereas AOB community structure was mainly associated with pH and salinity, indicating clear differences in environmental response patterns between the two groups.

### 3.4. Genus-Level Composition of AOA and AOB in Bohai Seawater

**Figure 5:**
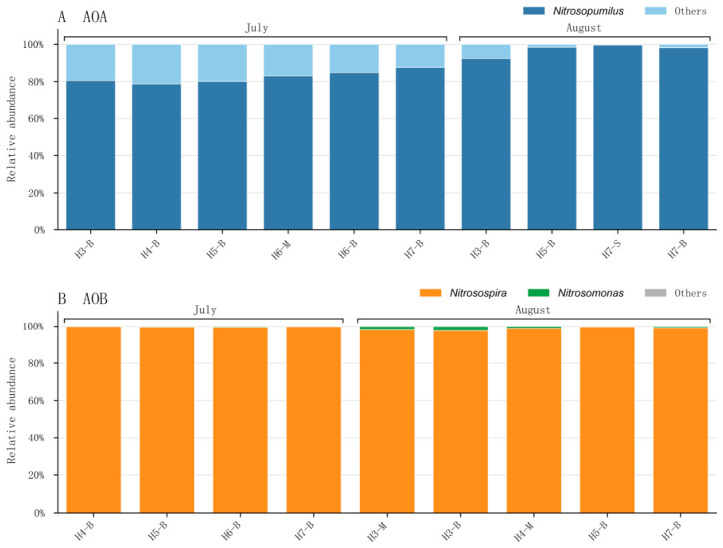
Genus-level relative abundances of AOA and AOB in Bohai Sea seawater samples. (**A**) shows the relative abundance of AOA genera, and (**B**) shows the relative abundance of AOB genera. Samples are grouped by month; only genera with a relative abundance ≥ 1% are shown; taxa < 1% and reads classified as unclassified/unidentified are pooled into “Others”.

At the genus level, the seawater AOA community was overwhelmingly dominated by *Nitrosopumilus* in all samples (Figure 5A), with relative abundances ranging from 78.8% to 99.6%. In the July samples, the relative abundance of *Nitrosopumilus* was relatively stable, ranging from 78.8% to 87.7%. In August, however, its relative abundance increased further to 92.5–99.6%, indicating clear month-related variation in the genus-level composition of AOA. Correspondingly, the relative abundance of the remaining AOA groups decreased in August. In the July bottom-water samples, the relative abundance of *Nitrosopumilus* also showed an increasing trend from station H3 to H7, whereas the proportion of the other AOA groups decreased accordingly.

By contrast, the seawater AOB community was dominated by *Nitrosospira* in all samples (Figure 5B), and its relative abundance remained above 97.5%. In the July samples, the genus-level composition of AOB was highly uniform, with *Nitrosospira* accounting for nearly all sequences and *Nitrosomonas* representing only a very small fraction. In August, the relative abundance of *Nitrosomonas* increased slightly in several samples, particularly in H3-M and H3-B, but *Nitrosospira* still remained overwhelmingly dominant. Overall, the month-related variation in genus-level composition was smaller for AOB than for AOA.

### 3.5. Standardized Abundance Patterns of Dominant AOA and AOB OTUs

**Figure 6:**
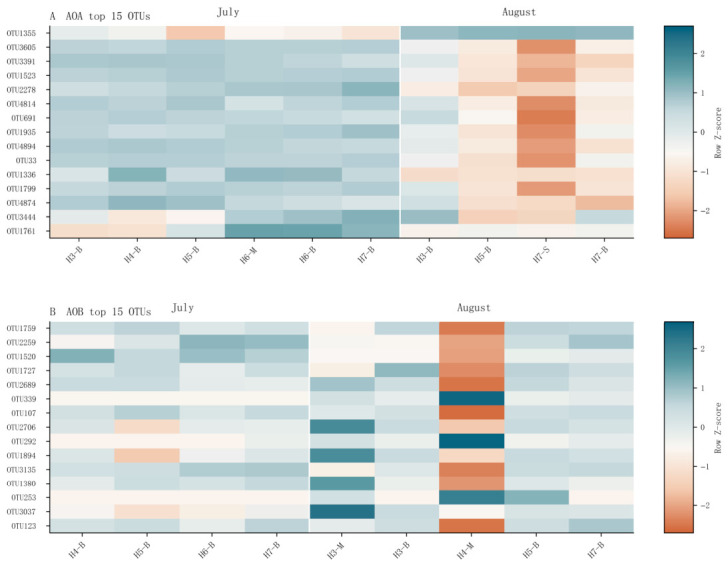
Heatmaps showing the standardized abundance patterns of the top 15 OTUs of AOA and AOB in Bohai Sea seawater samples. (**A**) shows the top 15 AOA OTUs and (**B**) shows the top 15 AOB OTUs. Samples are arranged by month (July and August), station, and water layer. Color intensity represents the row Z-score of log-transformed relative abundance for each OTU, highlighting month-dependent redistribution patterns among dominant OTUs.

To further identify the dominant taxa underlying community separation, the top 15 OTUs were examined using standardized abundance heatmaps (Figure 6). The dominant AOA OTUs showed clearer redistribution patterns between July and August. Among the top 15 AOA OTUs, 14 showed overall higher standardized abundance in July than in August, whereas only one OTU (OTU1355) was relatively enriched in August. These dominant OTUs were mainly affiliated with *Nitrosopumilus* and related *thaumarchaeotal lineages*, indicating that the month-related separation of AOA was not driven by a single dominant OTU, but rather by coordinated changes among multiple core OTUs. This pattern was consistent with the ordination analysis showing significant month-related separation of AOA communities (PERMANOVA, *p* < 0.01).

In contrast, the top 15 AOB OTUs showed comparatively more homogeneous patterns and weaker month-related redistribution. All top 15 AOB OTUs were affiliated with *Nitrosospira*. Among them, nine OTUs showed relatively higher standardized abundance in July, whereas six showed relatively higher abundance in August, suggesting weaker temporal turnover in AOB and a pattern dominated by relative replacement among a few abundant OTUs rather than overall community reorganization. This was also consistent with the PCoA result showing no significant month effect for AOB community structure (PERMANOVA, *p* = 0.157).

Overall, AOA exhibited stronger OTU-level temporal reorganization than AOB, further supporting that seawater AOA responded more strongly than AOB to summer temporal variation in the Bohai Sea.

## 4. Discussion

### 4.1. Seasonal Variability of Key Physicochemical Parameters in Bohai Sea Waters

The Bohai Sea water column showed a typical summer progression from June to August, characterized by increasing temperature, intensified water-column stratification, and enhanced bottom-water oxygen depletion (Figure 1). The vertical contrast in dissolved oxygen was most pronounced in August, when the difference between the bottom water and the surface and middle layers became larger, indicating weaker vertical exchange of water in the late summer. This pattern is consistent with the general mechanism of summer hypoxia formation in the Bohai Sea, in which the development of the thermocline and density stratification restricts downward oxygen supply from the surface, while continuous oxygen consumption associated with organic matter decomposition and respiration promotes bottom-water oxygen depletion [2,30,31]. In addition, salinity was generally lower in August than in June and July, and inorganic nitrogen distributions also shifted among water layers (Figure 1 and Figure 2), indicating that summer environmental change in the Bohai Sea was not limited to declining bottom-water DO, but also involved concurrent changes in temperature, salinity, and nutrient distributions. Compared with more stable open-ocean low-oxygen systems, the Bohai Sea is a semi-enclosed shallow sea with weak water exchange and is therefore more likely to develop such coupled changes driven by stratification, bottom-water oxygen consumption, and material redistribution [32,33].

### 4.2. Species Composition of AOA and AOB in Seawater

At the genus level, the seawater AOA community in the Bohai Sea was consistently dominated by *Nitrosopumilus*, whereas the AOB community remained dominated by *Nitrosospira* (Figure 5A,B). This trend reveals that dominant genera of ammonia-oxidizing communities were not fully replaced as summer hypoxia developed. Instead, these communities underwent internal reorganization, while their dominant composition remained stable. For AOA, the persistent dominance of *Nitrosopumilus* indicates that the archaeal ammonia oxidizers in Bohai seawater were mainly composed of typical marine lineages. Numerous prior studies reported that Thaumarchaeota (especially marine AOA lineages) are widely found in coastal and oxygen-deficient marine habitats and frequently dominate seawater AOA assemblages [34,35,36]. Therefore, the continued dominance of *Nitrosopumilus* in the present study is well explained by the environmental characteristics of the Bohai Sea as a semi-enclosed shallow sea with recurrent summer low oxygen. At the same time, Figure 6 shows clear redistribution among several dominant AOA OTUs between July and August, indicating that the main response of AOA occurred at the OTU level within marine-dominated lineages rather than through replacement at the genus level.

In contrast, AOB exhibited a far simpler genus-level composition. Nearly all samples in July were dominated by *Nitrosospira*, whereas *Nitrosomonas* appeared only at low relative abundance in a few samples in August (Figure 5B). Previous studies have suggested that *Nitrosospira* is more common in relatively high-salinity or marine environments, whereas *Nitrosomonas* is more likely to occur under lower-salinity conditions or in environments more strongly influenced by terrestrial inputs [37,38,39]. Therefore, the slight increase in *Nitrosomonas* in August was probably associated with the seasonal decline in salinity (Figure 1D–F), although this change was not sufficient to alter the overwhelming dominance of *Nitrosospira*. Overall, compared with AOA, the seawater AOB community showed stronger compositional conservatism, with only limited adjustment within the dominant-genus framework rather than obvious taxonomic replacement.

### 4.3. Community Distribution Patterns of AOA and AOB in Bohai Seawater

The present study showed that seawater AOA in the Bohai Sea responded more strongly to temporal change than AOB (Figure 4A). The AOA communities were clearly separated between July and August, and the effect of sampling month was significant (PERMANOVA, *p* = 0.009). This separation was not driven by a single OTU, but by the coordinated redistribution of multiple dominant AOA OTUs between the two months (Figure 6). Therefore, the month-related separation of seawater AOA was reflected not only in community structure but also in the reorganization of dominant taxonomic assemblages. Similar patterns have also been reported in coastal environments, where AOA communities are generally more likely than AOB communities to undergo seasonal reorganization [17,40,41]. In addition, studies in low-oxygen waters have shown that AOA communities can differ markedly among regions, whereas differences among water layers within the same low-oxygen system are not always significant. This suggests that AOA are usually more sensitive to temporal progression and the associated environmental changes, whereas vertical position itself may not be the primary factor structuring their communities [41].

In contrast, the month effect on AOB was weaker (Figure 4B). AOB samples were largely intermingled in the ordination space, the effect of sampling month was not significant (PERMANOVA, *p* = 0.157), and the standardized abundance patterns of the top 15 dominant OTUs were also broadly similar (Figure 6). These results indicate that the AOB community did not undergo a consistent month-related reorganization during the development of summer hypoxia in the Bohai Sea, but instead showed only local fluctuations against an overall stable background. Although a few samples, especially H4M826 in August, were separated from the main cluster, a more conservative interpretation is that this sample-level deviation was caused by short-term local physical disturbance or local hydrochemical variation, rather than by an overall restructuring of the AOB community. Previous studies have likewise shown that, under low-oxygen conditions, the abundance and diversity of AOA are often higher than those of AOB, indicating that AOA are generally the more sensitive and more dominant ammonia oxidizers under oxygen depletion [42,43,44]. In other words, although vertical stratification was clearly present in the environment, its effect on community structure was weaker than that of month-to-month variation; this pattern was especially evident for AOA, whereas it did not form a stable pattern in AOB.

### 4.4. Major Environmental Factors Driving the Differentiation of AOA and AOB Communities in Bohai Seawater

Environmental fitting analysis showed that the differentiation of AOA and AOB communities was associated with different environmental variables (Figure 4C). For AOA, temperature showed the strongest association with community structure, salinity was also significant, and NH_4_^+^ showed a marginal association, whereas DO and the other nutrient variables had much weaker explanatory power. Similar patterns have been reported in other coastal and estuarine waters. For example, AOA communities in the Pearl River Estuary were found to be highly sensitive to temperature variation and exhibited pronounced succession along salinity gradients, indicating that seasonal hydrographic changes strongly influence archaeal ammonia oxidizers [45]. These findings are consistent with the present study, suggesting that the temporal differentiation of AOA communities in the Bohai Sea was associated with the combined effects of seasonal environmental variation rather than oxygen depletion alone [45,46].

For AOB, pH and salinity were significantly associated with community structure (Figure 4C), whereas temperature, DO, NH_4_^+^, and the other nutrient variables showed no significant associations. Similar relationships have also been reported in estuarine and coastal ecosystems. Previous studies have shown that salinity is an important factor associated with AOB community composition, and increasing salinity is often accompanied by shifts in AOB diversity and community structure [47]. Likewise, studies from the Nanliu River Estuary and the Changjiang Estuary have reported significant associations between AOB communities and environmental variables, including salinity, pH, and ammonium, highlighting the combined influence of hydrochemical conditions on AOB community composition [37,39,48]. In the present study, despite the significant associations with pH and salinity, AOB communities showed no significant month effect and remained overwhelmingly dominated by *Nitrosospira*, with only a slight increase in *Nitrosomonas* in several August samples. Consistently, the dominant OTUs exhibited only limited redistribution between July and August. These observations suggest that seasonal environmental variation was associated with relatively modest adjustments within the dominant AOB lineage, rather than broad-scale community reorganization.

### 4.5. Niche Differentiation Between AOA and AOB During the Development of Summer Hypoxia in the Bohai Sea

Seawater AOA and AOB in the Bohai Sea showed different ecological strategies during the development of summer hypoxia. The diversity, community structure, and dominant OTU composition of AOA all changed markedly with sampling month, whereas AOB showed stronger conservatism at both the genus and OTU levels, with only slight variation in a few samples. This indicates that, under the background of summer hypoxia in the Bohai Sea, AOA were more readily filtered by environmental change than AOB and adapted to changing conditions through rapid adjustment within dominant lineages. Recent studies also support this pattern, showing that marine AOA can remain active in ammonia oxidation under oxygen depletion and may achieve finer niche differentiation through different substrate-use strategies [12,13]. This difference in response suggests that ammonia oxidation in Bohai seawater does not simply decline during hypoxia development, but is more likely maintained by AOA lineages that are better adapted to seasonal progression and oxygen depletion. Therefore, studies of nitrogen cycling in hypoxic areas of the Bohai Sea should not focus only on nitrogen-loss processes such as denitrification and anaerobic ammonium oxidation, but should also pay attention to water-column ammonia oxidizers, especially AOA, because of their potential roles in sustaining nitrification, redistributing substrates, and contributing to N_2_O formation [49,50].

The contrasting responses of AOA and AOB observed in this study may also have implications for evaluating seasonal hypoxia in coastal waters. Compared with AOB, AOA showed a more pronounced temporal response during hypoxia development, indicating that archaeal communities may provide additional information on environmental change in seasonally deoxygenated systems. Similar patterns have been reported in other coastal environments, where ammonia-oxidizing microorganisms responded differently to changes in oxygen availability and water-column stratification [7,21]. Because ammonia oxidation supplies substrates for downstream nitrogen transformation processes, shifts in ammonia-oxidizing communities may also influence nitrogen cycling under coastal deoxygenation [49]. Integrating microbial community information with routine environmental monitoring may therefore improve our understanding of ecological changes during seasonal hypoxia formation [51].

## 5. Conclusions

This study revealed that AOA and AOB exhibited distinct responses as oxygen depletion progressed in the Bohai Sea throughout summer. Low biomass in most surface layers precluded the identification of clear vertical stratification patterns for AOA and AOB. Temporal variation dominated AOA dynamics from June to August. AOA communities experienced substantial restructuring from July to August, alongside synchronous shifts in dominant OTUs. In contrast, AOB showed strong stability across temporal and spatial gradients. At the genus level, the archaeal and bacterial communities were consistently dominated by *Nitrosopumilus* and *Nitrosospira*, respectively. Their environmental associations also differed. Variation in the archaeal community was mainly driven by temperature and salinity, with a weak link to ammonium, while bacterial community structure was primarily linked to pH and salinity. These patterns indicate that the observed community changes reflected the combined effects of seasonal environmental variation rather than a direct response to declining oxygen alone.

Overall, the results demonstrate that microorganisms responsible for the same step of nitrogen cycling can follow markedly different ecological response patterns under rapidly changing coastal conditions. The stronger temporal reorganization of AOA suggests that they represent a more responsive component of the ammonia-oxidizing community during seasonal deoxygenation, with potential consequences for nitrification and substrate supply to downstream nitrogen transformations. Further studies integrating broader temporal sampling, absolute abundance measurements, and functional activity analyses will be needed to determine how these community-level changes translate into changes in ammonia oxidation rates and nitrogen cycling in the Bohai Sea.

## Data Availability

All data that support the findings of this study are contained in the main text and Supporting Information. The sequences have been submitted to the NCBI database (PRJNA1394680 and PRJNA1394683).

## Supplementary Materials

The following supporting information can be downloaded at: https://www.mdpi.com/article/10.3390/biology15151280/s1. Table S1: Summary of AOA and AOB sequencing information in Bohai seawater samples.

## References

[1] Guo X., Bednaršek N., Wang H., Feely R.A., Laurent A. (2022). Acidification and hypoxia in marginal seas. Front. Mar. Sci..

[2] Zhu Z.Y., Zhang J., Wu Y., Zhang Y.Y., Lin J., Liu S.M. (2011). Hypoxia off the Changjiang (Yangtze River) Estuary: Oxygen depletion and organic matter decomposition. Mar. Chem..

[3] Shakoor A., Ashraf F., Shakoor S., Mustafa A., Rehman A., Altaf M.M. (2020). Biogeochemical transformation of greenhouse gas emissions from terrestrial to atmospheric environment and potential feedback to climate forcing. Environ. Sci. Pollut. Res..

[4] Anand A., Garg S., Kashyap U., Arora P. (2025). Alteration in nitrogen cycle and its contribution to climate change: A review. Proc. Indian Natl. Sci. Acad..

[5] Yoshikawa C., Abe H., Aita M.N., Breider F., Kuzunuki K., Toyoda S., Ogawa N.O., Suga H., Ohkouchi N., Danielache S.O. (2016). Insight into nitrous oxide production processes in the western North Pacific based on a marine ecosystem isotopomer model. J. Oceanogr..

[6] Bristow L., Callbeck C., Larsen M., Altabet M., Dekaezemacker J., Forth M., Gauns M., Glud R., Kuypers M., Lavik G. (2017). N_2_ production rates limited by nitrite availability in the Bay of Bengal oxygen minimum zone. Nat. Geosci..

[7] Chen Q., Tang K., Zhai W., Zhu Z., Yang J.Y.T., He Z., Li M., Kao S.J., Yang J., Zheng Q. (2025). Microbial responses to ocean deoxygenation: Revisiting the impacts on organic carbon cycling. iScience.

[8] Pajares S., del Carmen Pelayo M. (2025). Phylogenetic diversity and distribution patterns of ammonia-oxidizing microorganisms in marine environments. Hydrobiologia.

[9] Tan Q., Zhu Y., Zhao Y., Zheng L., Wang X., Xing Y., Wu H., Tian Q., Zhang Y. (2025). Comparative analysis of niche adaptation strategies of AOA, AOB, and comammox along a gate-controlled river-estuary continuum. Water Res..

[10] Okabe S., Nukada K., Horiguchi K., Oshiki M. (2026). Oxygen scavenging enables microoxic survival of the marine anammox bacterium *Scalindua* sp.. ISME J..

[11] Sheikh S.M., Hussain U.M., Umare P.D., Jethani D.L., Prasad S.K. (2025). Marine archaea and their functional networks in oceanic biogeochemical cycling. Discov. Oceans.

[12] Hernández-Magaa E., Kraft B. (2024). Nitrous oxide production and consumption by marine ammonia-oxidizing archaea under oxygen depletion. Front. Microbiol..

[13] Stuehrenberg J., Kitzinger K., von Arx J.N., Graf J.S., Lavik G., Littmann S., Milucka J., Orsi W.D., Schorn S., Speth D.R. (2025). Urea use drives niche separation between dominant marine ammonia oxidizing archaea. Nat. Commun..

[14] Rabalais N., Diaz R.J., Levin L., Turner R.E., Gilbert D., Zhang J. (2010). Dynamics and distribution of natural and human-caused hypoxia. Biogeosciences.

[15] Okafor C., Bello A. (2026). Recent advances in marine nitrogen cycling: From diazotrophy to denitrification. Trop. Oceanogr. Res. Front..

[16] Gui X., Wang W., Qin D., Luo H., Qin F., Li K., Weng H., Zhang C. (2025). Revisiting the microbial nitrogen-cycling network: Bibliometric analysis and recent advances. Environ. Earth Sci..

[17] Vipindas P.V., Jabir T., Jasmin C., Balu T., Rehitha T.V., Adarsh B.M., Nair S., Abdulla M.H., Abdulaziz A. (2018). Diversity and seasonal distribution of ammonia-oxidizing archaea in the water column of a tropical estuary along the southeast Arabian Sea. World J. Microbiol. Biotechnol..

[18] Zhang J., Yang W., Song G., Zhang H., Zhao L. (2023). Summer bottom oxygen depletion dynamics and the associated physical structure in the Bohai Sea. Front. Mar. Sci..

[19] Zhao H.D., Kao S.J., Zhai W.D., Zang K.P., Zheng N., Xu X.M., Huo C., Wang J.Y. (2017). Effects of stratification, organic matter remineralization and bathymetry on summertime oxygen distribution in the Bohai Sea, China. Cont. Shelf Res..

[20] Guo J., Ji D., Zheng X., Li Y., Tang H., Hou C. (2024). Stratification in central Bohai Sea and how it has shaped hypoxic events in summer. Acta Oceanol. Sin..

[21] Han Y., Zhang M., Chen X., Zhai W., Tan E., Tang K. (2022). Transcriptomic evidences for microbial carbon and nitrogen cycles in the deoxygenated seawaters of Bohai Sea. Environ. Int..

[22] Jiang Y., Lou X., Wang M., Zheng M., Wang Z., Chen H. (2025). Genetic and transcriptional profiles of ammonia oxidizing communities in Bohai sediments: Abundance, activity, and environmental correlations. Front. Microbiol..

[23] Li M., He H., Mi T., Zhen Y. (2022). Spatiotemporal dynamics of ammonia-oxidizing archaea and bacteria contributing to nitrification in sediments from Bohai Sea and South Yellow Sea, China. Sci. Total Environ..

[24] Park H.D., Wells G.F., Bae H., Criddle C.S., Francis C.A. (2006). Occurrence of ammonia-oxidizing archaea in wastewater treatment plant bioreactors. Appl. Environ. Microbiol..

[25] Rotthauwe J.H., Witzel K.P., Liesack W. (1997). The ammonia monooxygenase structural gene amoA as a functional marker: Molecular fine-scale analysis of natural ammonia-oxidizing populations. Appl. Environ. Microbiol..

[26] Zhao R., Li X., Bei S., Li D., Li H., Christie P., Bender S.F., Zhang J. (2021). Enrichment of *nosZ*-type denitrifiers by arbuscular mycorrhizal fungi mitigates N_2_O emissions from soybean stubbles. Environ. Microbiol..

[27] Chen S., Zhou Y., Chen Y., Gu J. (2018). fastp: An ultra-fast all-in-one FASTQ preprocessor. Bioinformatics.

[28] Lu H., Chandran K. (2010). Factors promoting emissions of nitrous oxide and nitric oxide from denitrifying sequencing batch reactors operated with methanol and ethanol as electron donors. Biotechnol. Bioeng..

[29] Edgar R.C. (2013). UPARSE: Highly accurate OTU sequences from microbial amplicon reads. Nat. Methods.

[30] Zhou J., Zhu Z.Y., Hu H.T., Zhang G.L., Wang Q.Q. (2021). Clarifying water column respiration and sedimentary oxygen respiration under oxygen depletion off the Changjiang Estuary and adjacent East China Sea. Front. Mar. Sci..

[31] Wang H., Hu X., Wetz M.S., Hayes K.C. (2018). Oxygen consumption and organic matter remineralization in two subtropical, eutrophic coastal embayments. Environ. Sci. Technol..

[32] Wu M., Sun J., Shi L., Guo J., Morovati K., Lin B., Li Y. (2024). Vertical water renewal and dissolved oxygen depletion in a semi-enclosed Sea. J. Hydrol..

[33] Xing C., Cao X., Li H. (2025). On mechanisms controlling the interannual variability of the summertime bottom low-oxygen zones in the southern Bohai Sea. J. Geophys. Res. Oceans.

[34] Zou D., Liu H., Li M. (2020). Community, distribution, and ecological roles of estuarine archaea. Front. Microbiol..

[35] Li Y., Chen J., Lin Y., Zhong C., Jing H., Liu H. (2024). Thaumarchaeota from deep-sea methane seeps provide novel insights into their evolutionary history and ecological implications. Microbiome.

[36] Semedo M., Lopes E., Baptista M.S., Oller-Ruiz A., Gilabert J., Tomasino M.P., Magalhães C. (2021). Depth profile of nitrifying archaeal and bacterial communities in the remote oligotrophic waters of the North Pacific. Front. Microbiol..

[37] Gao J., Hou L., Zheng Y., Liu M., Yin G., Yu C., Gao D. (2018). Shifts in the community dynamics and activity of ammonia-oxidizing prokaryotes along the Yangtze estuarine salinity gradient. J. Geophys. Res. Biogeosci..

[38] Li M., Wei G., Shi W., Sun Z., Li H., Wang X., Gao Z. (2018). Distinct distribution patterns of ammonia-oxidizing archaea and bacteria in sediment and water column of the Yellow River estuary. Sci. Rep..

[39] Zhao M., Tang X., Sun D., Hou L., Liu M., Zhao Q., Klümper U., Quan Z., Gu J.-D., Han P. (2021). Salinity gradients shape the nitrifier community composition in Nanliu River Estuary sediments and the ecophysiology of comammox *Nitrospira inopinata*. Sci. Total Environ..

[40] Yang A., Zhang X., Agogué H., Dupuy C., Gong J. (2015). Contrasting spatiotemporal patterns and environmental drivers of diversity and community structure of ammonia oxidizers, denitrifiers, and anammox bacteria in sediments of estuarine tidal flats. Ann. Microbiol..

[41] He H., Zhen Y., Mi T., Fu L., Yu Z. (2018). Ammonia-oxidizing archaea and bacteria differentially contribute to ammonia oxidation in sediments from adjacent waters of Rushan Bay, China. Front. Microbiol..

[42] Abell G.C., Banks J., Ross D.J., Keane J.P., Robert S.S., Revill A.T., Volkman J.K. (2011). Effects of estuarine sediment hypoxia on nitrogen fluxes and ammonia oxidizer gene transcription. FEMS Microbiol. Ecol..

[43] La Cono V., La Spada G., Arcadi E., Placenti F., Smedile F., Ruggeri G., Michaud L., Raffa C., De Domenico E., Sprovieri M. (2013). Partaking of Archaea to biogeochemical cycling in oxygen-deficient zones of meromictic saline Lake Faro (Messina, Italy). Environ. Microbiol..

[44] Wang J., Kan J., Zhang X., Xia Z., Zhang X., Qian G., Miao Y., Leng X., Sun J. (2017). Archaea dominate the ammonia-oxidizing community in deep-sea sediments of the Eastern Indian Ocean—From the equator to the Bay of Bengal. Front. Microbiol..

[45] Ma L., Tan S., Liu H., Kao S.J., Dai M., Yang J.Y.T. (2021). Distribution and activity of ammonia-oxidizers on the size-fractionated particles in the pearl river estuary. Front. Mar. Sci..

[46] Zou D., Chen J., Zhang C., Kao S.-J., Liu H., Li M. (2023). Diversity and salinity adaptations of ammonia oxidizing archaea in three estuaries of China. Appl. Microbiol. Biotechnol..

[47] Bernhard A.E., Donn T., Giblin A.E., Stahl D.A.J.E.M. (2005). Loss of diversity of ammonia-oxidizing bacteria correlates with increasing salinity in an estuary system. Environ. Microbiol..

[48] Zheng Y., Hou L., Newell S., Liu M., Zhou J., Zhao H., You L., Cheng X. (2014). Community dynamics and activity of ammonia-oxidizing prokaryotes in intertidal sediments of the Yangtze Estuary. Appl. Environ. Microbiol..

[49] Lam P., Kuypers M.M. (2011). Microbial nitrogen cycling processes in oxygen minimum zones. Annu. Rev. Mar. Sci..

[50] Santoro A.E., Buchwald C., McIlvin M.R., Casciotti K.L. (2011). Isotopic signature of N_2_O produced by marine ammonia-oxidizing archaea. Science.

[51] Roman M.R., Altieri A.H., Breitburg D., Ferrer E., Gallo N.D., Ito S.-I., Limburg K., Rose K., Yasuhara M., Levin L.A. (2024). Reviews and syntheses: Biological indicators of low-oxygen stress in marine water-breathing animals. Biogeosciences.

