# Supplementary material for: Different Responses of Ammonia-Oxidizing Archaea and Bacteria to Oxygen Depletion During Niche Separation in Water Column of Bohai Sea
Source: Biology (Basel). 2026 Aug 4;15(15):1280. doi: 10.3390/biology15151280 (PMC13466061; doi:10.3390/biology15151280)
Supplement: Supplementary file 1 [file biology-15-01280-s001.zip › biology-4430868-supplementary.pdf]

In total, 840,800 AOA reads and 220,912 AOB reads were obtained, and the total numbers of OTUs were 1263 for AOA and 1031 for AOB. The distributions of AOA and AOB were not fully consistent among samples: both groups were detected in some samples, such as H5–B and H6–B in July, whereas only one group was detected in others, such as H3–B in July and H3–M in August. The samples with the highest sequence abundance for both AOA and AOB were collected in August. The highest AOA read number was detected in the bottom-water sample from station H5 (H5–B; 118,698 reads), whereas the highest AOB read number was detected in the middle-water sample from station H3 (H3–M; 41,334 reads). In samples where both AOA and AOB were detected, the number of AOA OTUs was higher than that of AOB in most cases. However, in the August sample H5–B, both the OTU number and observed species of AOB exceeded those of AOA. By contrast, in the August sample H7–B, the OTU number of AOA was higher than that of AOB, whereas its observed species was lower than that of AOB (Table S1).

Table S1 Summary of AOA and AOB sequencing information in Bohai seawater samples

Time	Station	Depth (m)	Sample Name	Sequences		OTUs		Observed species	
				AOA	AOB	AOA	AOB	AOA	AOB
16th June	H3	2	H3-S	-	-	-	-	-	-
		11	H3-M	-	-	-	-	-	-
		19	H3-B	-	-	-	-	-	-
	H4	2	H4-S	-	-	-	-	-	-
		11	H4-M	-	-	-	-	-	-
		23	H4-B	-	-	-	-	-	-
	H5	2	H5-	-	-	-	-	-	-
		12	H5-M	-	-	-	-	-	-
		25	H5-B	-	-	-	-	-	-
	H6	2	H6-S	-	-	-	-	-	-
		12	H6-M	-	-	-	-	-	-
		20	H6-B	-	-	-	-	-	-
	H7	2	H7-S	-	-	-	-	-	-
		12	H7-M	-	-	-	-	-	-
		20	H7-B	-	-	-	-	-	-
19th July	H3	2	H3-S	-	-	-	-	-	-
		11	H3-M	-	-	-	-	-	-
		19	H3-B	118462	-	597	-	392	-
	H4	2	H4-S	-	-	-	-	-	-
		11	H4-M	-	-	-	-	-	-
		23	H4-B	104552	23081	676	385	477	333
	H5	2	H5-S	-	-	-	-	-	-
		12	H5-M	-	-	-	-	-	-
		25	H5-B	63422	17372	618	353	556	344
	H6	2	H6-S	-	-	-	-	-	-
		12	H6-M	53568	-	500	-	481	-
		22	H6-B	48946	24670	513	372	513	314
	H7	2	H7-S	-	-	-	-	-	-
		12	H7-M	-	-	-	-	-	-
		20	H7-B	55335	21111	472	358	443	319
	H3	2	H3-S	-	-	-	-	-	-
		11	H3-M	-	41334	-	556	-	399
		19	H3-B	77535	21387	378	357	310	315
	H4	2	H4-S	-	-	-	-	-	-
		11	H4-M	-	34409	-	203	-	180
		23	H4-B	-	-	-	-	-	-

26th August	H5	2	H5-S	-	-	-	-	-	-
		12	H5-M	-	-	-	-	-	-
		25	H5-B	118698	21363	314	451	214	400
	H6	2	H6-S	-	-	-	-	-	-
		12	H6-M	-	-	-	-	-	-
		22	H6-B	-	-	-	-	-	-
	H7	2	H7-S	95373	-	144	-	103	-
		12	H7-M	-	-	-	-	-	-
		20	H7-B	104909	16185	367	327	257	327
